# Effects of Diet Strategy and Nutrients on the Progression and Prevention of Diabetic Cardiomyopathy: A Narrative Review

**DOI:** 10.1002/fsn3.71669

**Published:** 2026-04-24

**Authors:** Wen‐hui Deng, Kai‐xuan Lin, Abdallah Iddy Chaurembo, Francis Chanda, Yuan Li, Li‐Dan Fu, Hao‐Dong Cui, Xin‐Yue Tong, Chi Shu, Han‐Bin Lin

**Affiliations:** ^1^ School of Pharmacy, Laboratory of Drug Discovery from Natural Resources and Industrialization, State Key Laboratory of Mechanism and Quality of Chinese Medicine Macau University of Science and Technology Taipa Macau China; ^2^ Zhongshan Institute for Drug Discovery, Shanghai Institute of Materia Medica Chinese Academy of Sciences Zhongshan Guangdong China; ^3^ Department of Cardiology Zhongshan Hospital of Traditional Chinese Medicine Affiliated to Guangzhou University of Traditional Chinese Medicine Zhongshan Guangdong China; ^4^ State Key Laboratory of Chemical Biology Shanghai Institute of Materia Medica, Chinese Academy of Sciences Shanghai China; ^5^ University of Chinese Academy of Sciences Beijing China; ^6^ College of Food Science Shenyang Agricultural University Shenyang Liaoning China

**Keywords:** diabetic cardiomyopathy, dietary intervention, inflammation, oxidative stress

## Abstract

Diabetic cardiomyopathy (DCM) has emerged significantly as a prevalent clinical burden since the 19th century. Currently, despite scarce evidence, dietary strategies are emerging as adjunctive measures to treat DCM. Among these dietary strategies, nutrients such as vitamin D, ω‐3 PUFAs, zinc, selenium, resveratrol, anthocyanins, and curcumin have been repeatedly associated with attenuating cardiac oxidative stress, inflammation, and fibrosis in rodent models. However, these results remain scarce and inconsistent in humans. On the contrary, caloric restriction frequently improves cardiac energetics and reduces inflammatory markers in both animals and small patient cohorts. Another form of dietary strategy is the ketogenic diet, which evokes divergent, time‐dependent effects. For instance, short‐term feeding enhances myocardial ketone utilization and calcium handling, whereas prolonged exposure has been linked to lipotoxicity, impaired Treg response, and interstitial fibrosis in diabetic mice. As such, these nuances suggest that despite encouraging findings, challenges, including limited clinical trials, individual differences in dietary responses, and patient adherence, require further investigation. Therefore, in this narrative review, we synthesize preclinical and clinical findings on how dietary strategies, including nutrients, bioactive compounds, and caloric restriction, modulate myocardial structure and function in diabetes. Emphasis is placed on distinguishing robust mechanistic insights from preliminary translational findings and on identifying priorities for future human research.

## Graphical abstract

Dietary strategies modulate the progression of diabetic cardiomyopathy by targeting oxidative stress, inflammation, mitochondrial dysfunction, and myocardial fibrosis. Specific nutrients and bioactive compounds, as well as caloric restriction, improve myocardial metabolism and redox balance, whereas ketogenic diets exert time‐dependent beneficial or adverse cardiac effects. These findings highlight diet‐based interventions as promising adjunctive approaches for diabetic cardiomyopathy, while underscoring the need for individualized and evidence‐guided implementation.

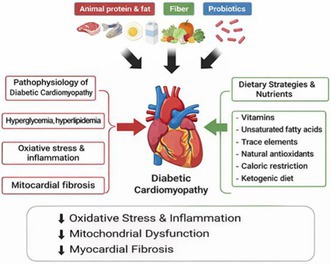

## 1 Introduction

Diabetes is a widespread chronic metabolic disease that leads to various complications and lowers life expectancy worldwide (Disease GBD, Injury and Risk Factor C 2025). According to the 10th International Diabetes Federation (IDF) Global Atlas Report, the diabetes population will exceed 783.2 million by 2045, with China having the highest number of people with diabetes, estimated at over 174 million in 2045 (Sun, Saeedi, et al. 2022). Diabetes usually increases the risk of cardiovascular complications, and the diagnosis of DCM is made by excluding other types of cardiomyopathy (Williams et al. 2017). DCM is characterized by early diastolic dysfunction of the heart in individuals with diabetes, in the absence of hypertension, coronary artery disease, or other cardiac risk factors. Furthermore, hyperglycemia and insulin resistance are independently linked to the development and progression of cardiac dysfunction and heart failure in these patients (Lorenzo‐Almoros et al. 2022).

Among 414,672 patients with type 2 diabetes mellitus (T2DM), 82.8% suffer from hyperlipidemia (Huang et al. 2022). In patients with diabetes mellitus, insulin resistance or deficiency, the diabetic heart relies more on free fatty acids (FFA) as the major substrate for oxidative phosphorylation. Concomitant hyperlipidemia shifts myocardial fuel preference toward free fatty acids (FFAs), causing lipotoxic metabolites (ceramides, diacylglycerol) to accumulate and further impair mitochondrial efficiency (Chong et al. 2017).

At present, due to the lack of effective methods in the treatment of DCM, oral hypoglycemic agents are clinically considered to inhibit the diabetic heart damage; however, the results are not satisfactory (Zhao et al. 2022; Galis et al. 2024; Murtaza et al. 2019). Therefore, in addition to the use of diabetes medications, lifestyle and medical nutrition therapy are being considered to improve the treatment of T2DM.

An appropriate dietary strategy has long been recognized as an effective way to promote health. Clinically, dietary strategies improve DCM not merely by lowering blood glucose, but by interrupting the pathophysiological cascade that hyperglycemia triggers in the myocardium. Studies have reported that small, frequent meals flatten post‐prandial glucose excursions, thereby reducing repetitive bursts of mitochondrial ROS that activate the NLRP3 inflammasome and precipitate sarcoplasmic‐reticulum Ca^2+^ leak, a key driver of diastolic dysfunction in DCM (Kim et al. 2025). Other studies have shown that ketogenic diets (KDs) shift myocardial substrate preference from glucose to ketone bodies, reduce the uptake and utilization of glucose by cardiomyocytes (Trang et al. 2023). Moreover, it diminishes AGE‐mediated cross‐linking of collagen and titin, which stiffens the diabetic left ventricle (Lee et al. 2023). Importantly, caloric restriction further restores AMPK‐mediated autophagy, clears lipotoxic ceramides and diacylglycerol, thereby rescuing mitochondrial efficiency and attenuating ROS production (Oza et al. 2021).

Also, beyond macronutrient composition, food‐derived bioactive compounds directly target the molecular lesions of DCM. For example, astragaloside IV (from astragalus) improves abnormal myocardial lipid metabolism (Wang et al. 2020), while mogrosides (from monk fruit) suppress the NF‐κB signaling pathway (Cai et al. 2021). In addition, dietary polysaccharides ameliorate DCM by suppressing endoplasmic reticulum stress, inhibiting cardiomyocyte apoptosis, and enhancing antioxidant defense (Jin et al. 2025). As such, aligning nutrient delivery and bioactive compounds integrates nutrition into the therapeutic modulation of DCM. However, dietary strategies are not universally applicable to all patients with diabetes. For instance, while a zinc‐supplemented diet may improve heart function in DCM patients with zinc deficiency, excessive supplementation may not benefit patients and could even disrupt the body's elemental homeostasis. Furthermore, it impairs the catalytic activity of Cu/Zn SOD and exacerbates oxidative stress (Das et al. 2025). Similarly, although a short‐term KD can mitigate diabetic heart injury, prolonging this dietary approach may lead to ketoacidosis and aggravate cardiac fibrosis (Abdurrachim et al. 2019; Tao et al. 2021). These conflicting findings suggest that despite the promising effect of dietary strategies, challenges, including limited clinical trials, individual differences in dietary responses, and patient adherence, require further investigation. Therefore, in this narrative review, we provide a non‐systematic synthesis of how vitamins, unsaturated fatty acids, trace elements, natural antioxidants, KDs, and caloric restriction modulate DCM in preclinical and clinical settings. The integration of this evidence will help clinicians and researchers decide when and when not to recommend specific nutrient or diet‐based strategies for diabetic patients with the risk of heart failure.

Based on the review purpose, the review synthesizes preclinical and clinical evidence on dietary strategies and specific nutrients in the context of DCM. The literature was identified by searching through PubMed and Web of Science from database inception to December 2025. The search strategy combined terms related to the condition (“diabetic cardiomyopathy”, “diabetic heart”, “diabetic cardiac dysfunction”) with dietary interventions (“diet”, “nutrition”, “caloric restriction”, “ketogenic diet”, “vitamin”, “unsaturated fatty acid”, “omega‐3”, “PUFA”, “trace element”, “zinc”, “selenium”, “resveratrol”, “anthocyanin”, “curcumin”).

Inclusion criteria encompassed (1) original research articles or reviews published in English; (2) studies investigating dietary components, nutrients, or dietary patterns (caloric restriction, KD) in animal models of diabetes or in human participants with diabetes and cardiac complications. Furthermore, administration in animals is limited to oral gavage and consumption of dietary components in drinking water; and (3) studies reporting cardiac structural, functional, or molecular outcomes relevant to DCM. Exclusion criteria included: non‐diabetes‐related cardiomyopathies, case reports/series with *n* < 5, editorials, and studies focused solely on glycemic control without cardiac endpoints.

## 2 The Role of Dietary Components in DCM

**FIGURE 1:**
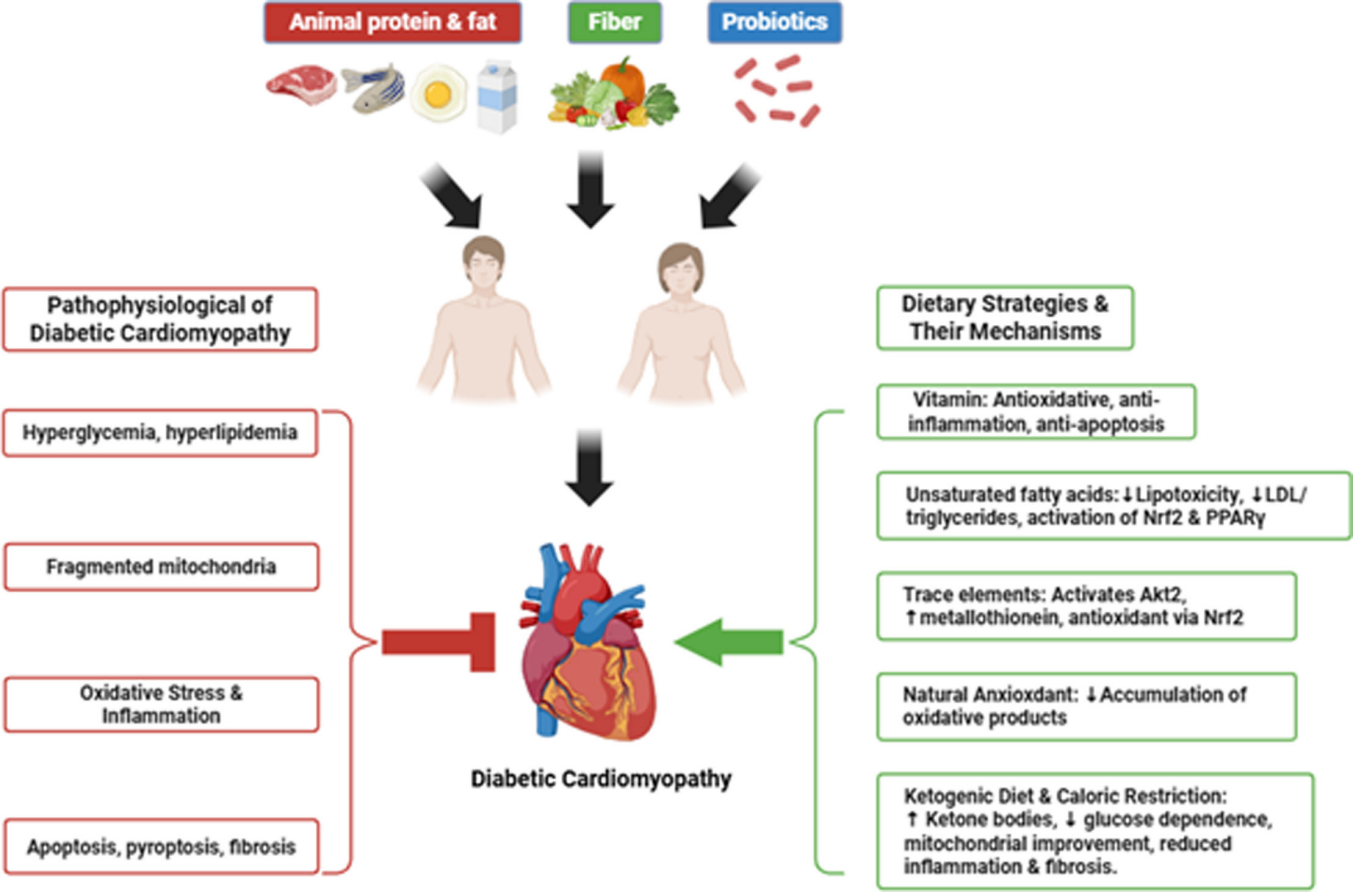
Improving diabetic cardiomyopathy by modulating dietary components and strategies. This figure depicts the interconnectedness of pathophysiological progression and DCM. Dietary components include vitamins, unsaturated fatty acids, trace elements, and natural antioxidants, as well as dietary strategies like ketogenic diet and caloric restriction can improve diabetic cardiomyopathy.

Nutrients play an important role in regulating physiological processes, and most nutrient sources form part of the daily diet. The maintenance of physiological well‐being is fundamentally dependent on the consistent consumption of a nutritionally complete and balanced diet. In addition to carbohydrates, fats, and proteins, diets also contain many substances, such as vitamins, trace elements, and other ingredients, which participate in biochemical reactions and metabolism. There are some special active ingredients in homologous food and medicine, which have therapeutic effects to a certain degree. Regular consumption of these foods is beneficial for human health and for the prevention and treatment of diseases (Figure 1).

### 2.1 Vitamin

Vitamins are organic substances necessary for maintaining normal physiological functions in humans and animals. Most of them cannot be produced by organisms themselves and must be ingested from the daily diet. Only a few vitamins can be synthesized in the body or produced by the gut microbiota. Increasing evidence supports the association between vitamins and T2DM. Moreover, vitamin intake from food was significantly associated with reduced mortality in US adults with diabetes (Liu, Cao, et al. 2024).

Vitamin D deficiency is highly prevalent in patients with diabetes. Notably, low serum 25‐hydroxyvitamin D (25(OH)D) levels are associated with an increased risk of cardiovascular complications, including DCM (Md Isa et al. 2023; Chen, Wan, et al. 2023). Although observational studies commonly link low serum 25(OH)D to worse cardiac outcomes, interventional evidence for cardiac protection primarily comes from preclinical models. For example, in HFD + STZ‐induced diabetic rats, gavage of 0.03 μg/kg/day vitamin D3 for 6 weeks reduced myocardial apoptosis and improved cardiac function by inhibiting the Fas/FasL pathway (Zeng et al. 2017). Moreover, oral 25(OH)D inhibits the nuclear translocation of forkhead box protein O1 (FoxO1) by activating vitamin D receptor (VDR). This, in turn, reduces the excessive autophagy activity attributed to diabetic heart and reduces cardiomyocyte apoptosis (Guo, Lin, et al. 2020). Furthermore, recent evidence has demonstrated that supplementation with 1,25(OH)2D3 alleviates DCM via suppressing pyroptosis through the NOX4/NLRP3 inflammasome pathway (Wang, Sun, et al. 2025). Notably, dietary intake and serum levels have distinct implications. For instance, dietary sources (e.g., fatty fish, egg yolks, fortified dairy) contribute to maintaining serum 25(OH)D while supplementation efficacy varies by individual factors (e.g., obesity, renal function, skin synthesis capacity). Clinically, for patients with diabetes at risk of DCM, regular monitoring of serum 25(OH)D is recommended. If deficiency is identified, dietary adjustment or vitamin D3 supplements (under medical supervision) may be considered to mitigate myocardial injury. While preclinical studies consistently demonstrate cardioprotective effects of vitamin D supplementation through multiple pathways (apoptosis inhibition (Zeng et al. 2017), autophagy attenuation (Guo, Lin, et al. 2020), pyroptosis suppression (Wang, Sun, et al. 2025)), human evidence remains largely observational. Moreover, interventional trials specifically targeting DCM are scarce, small, and often confounded by co‐interventions or short follow‐up. Large‐scale randomized controlled trials (RCTs) are urgently needed to determine whether vitamin D supplement provides meaningful cardiac benefit beyond conventional diabetes management.

Oxidative stress is a key mechanism in the development of DCM; therefore, enhancing antioxidative ability can also protect heart function in patients with diabetes. Vitamin E is a natural antioxidant, and dietary supplementation with vitamin E was sufficient to reduce myocardial 8‐*iso* PGF2α and GSSG levels which resulted in significant protection against cardiac dysfunction induced by high blood glucose (Hamblin et al. 2007). Vitamin C is also a natural antioxidant that can help improve DCM by mitigating oxidative stress (Mushtaq et al. 2021).

Similarly, Vitamin B12 prevents DCM by reducing ROS production (Hu et al. 2022; Kakoki et al. 2021). A key clinical consideration is metformin‐induced vitamin B12 deficiency: long‐term use of metformin impairs intestinal absorption of B12, with a prevalence of deficiency in T2DM patients (Chapman et al. 2016; de Jager et al. 2010). In addition, low serum vitamin B12 and folate levels are associated with a higher cardiovascular disease mortality in diabetes (Liu et al. 2022). Therefore, for T2DM patients on long‐term metformin (especially those with cardiac dysfunction or DCM risk), it is important to monitor serum B12 levels every 1–2 years. Deficiency can be corrected via dietary sources, including meat, dairy, eggs, or oral supplements (1000 μg), which may help mitigate myocardial oxidative stress and improve cardiac function (Chapman et al. 2016).

Under normal physiological conditions, the regulation of mitochondrial dynamics is a complex process involving different dynamin‐related GTPases that maintain a balance between mitochondrial fusion and fission. However, exposure to an excess nutrient environment, such as high fat and high‐glucose levels, promotes mitochondrial fission and decreases mitochondrial fusion, eventually resulting in mitochondrial dysfunction (Rovira‐Llopis et al. 2017). Mfn2 is a key regulator of mitochondrial fusion (Chandhok et al. 2018), and nicotinamide ribose, a derivative of vitamin B3, upregulates the expression of Mfn2 and improves heart function (Hu et al. 2022). Additionally, protein expression related to energy metabolism, such as glycolmetabolism‐related and lipid metabolism‐related protein AMPK, is impaired in diabetic hearts, and myocardial cells cannot handle excess glucose and fatty acids, resulting in the generation of extra ROS and toxic fatty metabolic products. Alpha‐lipoic acid is a vitamin analog rich in many animal organs. Alpha‐lipoic acid reduces toxic products through upregulating AMPK and improving cardiac glucose metabolism (Lee et al. 2012; Dugbartey et al. 2022). Other vitamins also have the potential to improve DCM. Although there are a few studies directly on DCM mice or patients, they also show heart benefits in patients with diabetes. Folic acid alleviates HFD‐induced pyroptosis by inhibiting the Hippo signaling pathway (Hong et al. 2021).

However, not all vitamins can protect the heart from diabetes. Vitamin A, also known as retinol, is a fat‐soluble vitamin predominantly found in animal‐derived foods, while its precursor, provitamin A, is primarily present in plant‐based foods (Takahashi et al. 2022). In preclinical models, excessive retinol intake (male mice: 800 IU per mouse, administered every 2 days; equivalent to approximately 7.8–8.5× the human tolerable upper intake level and 29–33× the human recommended dietary allowance) reduces all‐trans retinoic acid bioavailability, leading to lipotoxicity and ferroptosis, thereby exacerbating DCM (Wu et al. 2023). In contrast, moderate intake (10–20 μg/kg/day) does not induce cardiac damage. For humans, the recommended dietary allowance is 900 μg RE/day for men and 700 μg RE/day for women; excess intake (> 3000 μg RE/day, typically from supplements or high animal liver consumption) increases toxicity risk (Institute of Medicine (US) Panel on Micronutrients 2001), although direct links to human DCM are lacking. Moreover, several epidemiological investigations have found that dietary intake of β‐carotene and retinol is negatively associated with the risk of diabetes, especially in men (Su et al. 2022). However, human data on sex‐specific responses to vitamin A are limited, and further clinical research is needed to clarify the optimal doses for patients with diabetes.

In conclusion, most vitamins exert protective effects against diabetic myocardial damage in diabetes mellitus (Table 1). Furthermore, the intrinsic associations between vitamins and DCM should be elucidated. For instance, elucidating the correlation between vitamin deficiency and the risk of developing DCM is instrumental in formulating an appropriate vitamin dietary supplementation plan tailored to the patient's specific disease state.

**TABLE 1:** The role of vitamins on DCM.

Component	Research object	Method	Effect	Mechanism
Vitamin D3^a^ (Zeng et al. 2017)	SD rat, HFD + STZ induced	Gavage VD3 (0.03 μg/kg/day)/6 weeks	Blood glucose↓, CK↓, LDH↓, Fas↓, Fasl↓	Attenuate myocardial cell apoptosis
Vitamin D3^a^ (Guo, Lin, et al. 2020)	Zucker diabetic fatty rats and Zucker lean rats	Gavage (320 U/kg b w) once every 2 days	Blood glucose↓, CK‐MB↓, C‐cas3↓, Bax/Bal2↓, fibrotic area↓, Beclin‐1↓, LC3↓, p62↓, VDR↑, FoxO1↓	Attenuated autophagy
Vitamin D3^a^ (Wang, Sun, et al. 2025)	db/db mice	Gavage 1, 25(OH)_2_D_3_ (6 μg/kg bw)/17 weeks	Blood glucose↓, cardiomyocyte cross‐sectional area↓, LVEF↑, LVFS↑, E/A↑, NOX4↓, NLRP3↓, ASC↓, IL‐1β↓, IL‐18↓	Inhibiting pyroptosis
Nicotinamide riboside^a^ (Hu et al. 2022)	db/db mice	Gavage Nicotinamide riboside (400 mg/kg/day)/4 weeks	LVFS↑, LVEF↑, fibrotic area↓, HW/TL↓, ROS↓, MDA↓, cleaved‐caspase 3↓, Mfn2↑	Improve mitochondrial dysfunction
Vitamin B12^a^ (Kakoki et al. 2021)	Ins2^Akita/+^ mice	Vitamin B12 was provided through their drinking water (10 mg/kg)/12 weeks	LVEF↑, LVPWd↑, LVIDd↓, GSH/GSSG↑, fibrotic area↓, SAMe↑, DNMT↑, SOCS1/3↓, IGF‐1↑	Mitigate oxidative stress
Vitamin C^a^ (Mushtaq et al. 2021)	SD rats, alloxan induced	Oral gavage of ascorbic acid (80 mg/kg) for 11 days	Blood glucose↓, HW/BW, HW/TL, fibrosis area↓, GATA4↓, ROS↓, SOD↑, GSH↑, catalase↑, PGC‐1α↑, PPARα↑, Drp1↓, Mfn2↑	Mitigate oxidative stress
Vitamin E^a^ (Ojo et al. 2022)	Wistar rats, HFD + STZ induced	Oral gavage of 10 mg/kg vitamin E for 28 days	Blood glucose↓, TG↓, CH↓, lipid peroxidation↓, mitochondrial permeability transition pore opening↓, caspase 9↓, caspase 3↓	Attenuate myocardial cell apoptosis
Folic acid^a^ (Hong et al. 2021)	C57BL/6 mice HFD + STZ induced	8 mg/kg folic acid was given by gavage every 2 days	Blood glucose↓, NLRP3↓, ASC↓, cleaved‐caspase1↓, IL‐1β↓, IL‐18↓, p‐YAP↓, p‐LATS↓	Alleviates cell Pyroptosis
α‐lipoic acid^a^ (Dugbartey et al. 2022)	Male SD rats, nicotinamide + STZ induced	Gavage α‐lipoic acid (60 mg/kg/day)/6 weeks	HW/BW↓, HbA1c↓, insulin↑, HOMA‐β↑, CBS↑, CSE↑, 3‐MST↑, H_2_S↑	Activated cardiac H_2_S‐synthesizing enzymes

^a^ Interventional studies.

### 2.2 Unsaturated Fatty Acids

Fatty acids include saturated and unsaturated fatty acids. Unsaturated fatty acids are a class of fatty acids that contain one or more double bonds in their structure. Most unsaturated fatty acids cannot be synthesized by the human body and must be obtained through dietary intake. Beyond providing partial energy, they participate in biological actions, such as serving as essential components of phospholipids to maintain cell membrane fluidity, regulating inflammatory responses through metabolites of unsaturated fatty acids (e.g., eicosanoids), and modulating lipid profiles by reducing low‐density lipoprotein (LDL) and triglyceride levels while increasing high‐density lipoprotein (HDL) (Wiktorowska‐Owczarek et al. 2015).

Previous studies have shown that saturated fatty acids can increase the risk of systolic cardiac dysfunction (Haffar et al. 2015). One of the pathological mechanisms of DCM is excessive lipid accumulation, which leads to impaired myocardial oxidative stress function (Dirkx et al. 2011). At present, the most widely used unsaturated fatty acids are extracts from fish oil: eicosapentaenoic acid (EPA) and docosahexaenoic acid (DHA), which can reduce triglycerides and low‐density lipoprotein in blood and increase high‐density lipoprotein in both animal experiments and clinical studies; therefore, fish oil is widely recommended for protection against cardiovascular diseases (Yang et al. 2016). The mechanism of action of unsaturated fatty acids is that their oxidation and nitration products are electrophilic substances, which can induce PTM reactions (PTM modification) through enzyme and oxidation reactions, thus changing protein function and gene expression, such as activating Nrf2 and PPARγ and reducing the expression of NF‐κB (Delmastro‐Greenwood et al. 2014). Notably, a recent study revealed that EPA activates G protein‐coupled receptor 120 (GPR120) in macrophages. This led to upregulation of HO‐1 expression and drove macrophage polarization toward the antioxidant Mox phenotype, rather than the conventional M2 type. This shift effectively suppressed M1 macrophage‐induced cardiac oxidative stress and cardiomyocyte injury (Li et al. 2024).

The diets of ordinary people contain both saturated and unsaturated fatty acids (FAs). The intake of ω‐3 PUFAs, an unsaturated fatty acid, upregulates GUT4 and inhibits DPP4, significantly reduces blood glucose, and reduces the adverse effects of hyperglycemia, especially the abnormal rise of myocardial connexin‐43, which improves cardiac function (Anna et al. 2014), and DHA supplementation in the diet can decrease cardiomyopathy in obese rats induced by harmful saturated fatty acids and improve the response of heart mitochondria to palmitic acid (PA) by reducing oxidative stress and lipotoxicity (Gui et al. 2020).

In addition to fish oil, another food, krill oil, is also rich in DHA, and after 24 weeks of feeding krill oil, heart fibrosis and collagen deposition in diabetic mice were significantly improved by inhibiting cell pyroptosis (Sun, Sun, et al. 2022; Mayyas et al. 2018). Therefore, dietary supplementation with unsaturated fatty acids could help protect patients with diabetes from harmful saturated fatty acids and has the potential to improve metabolic syndrome and diseases related to abnormal glucose and lipid metabolism (Table 2).

**TABLE 2:** The role of unsaturated fatty acids on DCM.

Component	Research object	Method	Effect	Mechanism
ω‐3 PUFAs^a^ (Anna et al. 2014)	Wistar Kyoto rats, HFD + STZ induced	Gavageω‐3 PUFAs 100 mg/100 g/day for 4 weeks	blood glucose↓, EF↑, myh7↓, CX43↓, P‐CX43↓	Improve abnormal myocardial remodeling
DHA^a^ (Gui et al. 2020)	Sprague–Dawley rats fed HFD	High‐fat diet supplemented with DHA (20 μM) for 16 weeks	ROS↓, myh7↓, TNFα↓, IL6↓, fibrosis area↓, 4HNE↓, Tm20↑	Reduce oxidative stress and ameliorate lipid toxicity
EPA^a^ (Li et al. 2024)	C57 mice, HFD + STZ induced	Feed high‐fat containing 2% EPA	LVEF↑, LVFS↑, E/A ratio↑, GRP120↑, HO‐1↑, F4/80&HO‐1↑, F4/80& TrxR1↑	Promotes Mox polarization in monocyte derived macrophages
Fish oil^a^ (Mayyas et al. 2018)	Wistar rats, STZ‐induced	Gavage 4 μL/g body weight for 8 weeks	arachidonic acid↓, total nitrite↓, endothelin‐1↓, myeloperoxidase↓, SOD↓, TGF‐β1↓, P38↓, MMP2↓	Attenuated cardiac inflammation, oxidative stress and fibrosis
Krill Oil^a^ (Sun, Sun, et al. 2022)	C57 mice, HFD + STZ induced	1.5% KO‐containing high‐fat diet for 24 weeks	fibrotic area↓, iNOS↓, 8‐OHdg↓, GSDMD↓, SIRT3↑, PGC‐1α↑, NLRP3↑,	Inhibit pyroptosis

^a^ Interventional studies.

Despite robust preclinical data, RCTs of marine ω‐3 PUFAs in broad populations have largely returned neutral results (Liao et al. 2021; Liu, Liu, et al. 2024). For instance, in a study that included 15 meta‐analyses of ω‐3 PUFAs in cardiovascular disease, doses below 1 g/day (including 1 g/day and 2–4 g/day) showed no benefit. However, doses of 1.8 g (Yokoyama et al. 2007) and 4 g/day (Amarin Pharma Inc 2011) reduced the risk of cardiovascular events, suggesting a dose‐dependent effect (Goel et al. 2018). A meta‐analysis incorporating 12–13 studies showed that omega‐3 PUFA (EPA, DPA, DHA) were significantly associated with reduced risks of total cardiovascular disease (CVD), coronary heart disease (CHD), and total mortality, with mostly linear dose–response trends (every 1 standard deviation increase in circulating levels), except for the DHA‐CVD association, which was non‐linear (benefits emerging when DHA exceeds approximately 2% of total fatty acids) (Jiang et al. 2022). Higher levels of omega‐3 polyunsaturated fatty acids in the diet are consistently associated with lower cardiovascular mortality risk (Chen, Leng, et al. 2023). However, studies have explored the relationship between cardiovascular disease and the consumption of lean fish (rich in ω‐3 PUFAs) versus fatty fish (with lower PUFA content). It has been reported that fatty fish are inversely associated with coronary heart disease (CHD) (RR: 0.92; 95% CI: 0.86–0.97), CHD mortality (RR: 0.83; 95% CI: 0.70–0.98), and total mortality (RR: 0.97; 95% CI: 0.94–0.99), whereas no such associations have been observed for lean fish (Giosue et al. 2022). This indicates that no apparent benefit attributable to high ω‐3 PUFA content was observed.

Currently, clinical research on unsaturated fatty acids still has shortcomings. Firstly, the baseline EPA + DHA level (the “Omega‐3 Index”) of most participants is unknown. Once a steady state is reached, additional supplementation may confer little extra benefit. Furthermore, trial doses and serum concentrations are not equivalent. The differences in supplement formulation and dosage across studies can produce disparate serum levels. Finally, contemporary drug therapies (statins, ACEi/ARB, SGLT2 inhibitors) obscure any modest benefits of unsaturated fatty acids.

Further randomized controlled trials are necessary to comprehensively assess the effects of EPA, DHA, and ω‐3 PUFAs on cardiovascular outcomes and the underlying mechanisms involved. Current research on the effects of unsaturated fatty acids on DCM is limited, with few studies examining the underlying molecular mechanisms. Simultaneously, there is an inconsistency where unsaturated fatty acids show no significant benefits in clinical data but demonstrate potential improvements in diabetic cardiovascular effects in animal studies, which remains a critical issue that needs resolution. This may serve as the next research objective for unsaturated fatty acids.

### 2.3 Trace Elements

Trace elements play an essential role as components of some metabolic enzymes and participate in regulating ion channels and redox status (Ozturk et al. 2013). In recent years, many inorganic trace elements have been used as dietary supplements to alleviate metabolic damage in patients with diabetes. Zinc homeostasis is generally impaired in patients with diabetes. Zinc deficiency in patients with diabetes promotes the occurrence of diabetes complications, and zinc supplementation can help patients control their blood glucose levels and reduce the risk of complications (Barman and Srinivasan 2022). Metallothionein (MT) is a protein rich in cysteine that has a high affinity for zinc, and its expression is induced by the increase of zinc and plays an antioxidant role by scavenging free radicals; therefore, it can offset the inflammation, oxidative stress and myocardial remodeling caused by diabetes (Baltaci et al. 2018; Giacconi et al. 2017). Akt2 is a kinase that mediates multiple metabolic reactions, including the regulation of blood glucose and fatty acid metabolism. It is regulated by a negative regulator TRB3. Induction of TRB3 expression in MT cardiac‐specific transgenic (MT‐TG) mice reduced the expression level of Akt2 and the protective effect of MT protein on the heart. When the TRB3 gene was silenced in MT mice, the expression of Akt2 increased and improved the heart function of diabetic mice. This phenomenon was observed when a zinc‐supplemented diet was administered. MT protein maintains the myocardial protection of Akt2 by inhibiting TRB3 in a zinc‐supplemented diet (Gu et al. 2018). However, Akt2 is not an absolute regulator of MT proteins. In Akt2 gene knockout mice, supplementation with zinc or induction of MT protein expression can still improve glucose and lipid metabolism, indicating that MT protein has other downstream pathways. In Akt2 gene knockout mice with MT protein overexpression, the expression of ERK1/2 increased, suggesting that cardiac overexpression of MT may compensate for Akt2 deletion by activating the ERK pathway (Huang et al. 2021).

ROS overproduction and impaired antioxidant capacity are factors in the pathogenesis of DCM, and nuclear factor erythroid 2‐related factor 2 (Nrf2) can upregulate the expression of various antioxidant enzymes, including NADPH dehydrogenase (NQO1), superoxide dismutases (SODs), and glutathione peroxidases (GPX) (Howden 2013), which reduce the damage caused by oxidative stress to the myocardium. Several studies have shown that a variety of natural products can reduce heart dysfunction, myocardial hypertrophy, and myocardial fibrosis caused by diabetes by activating Nrf2 (Wei et al. 2022; Xu et al. 2016; Ying et al. 2018). In the diet of diabetic mice with different zinc levels, zinc deficiency decreases the expression of Nrf2 in the diabetic heart and lowers the downstream antioxidant factors, thus aggravating myocardial oxidative damage and inflammation; however, zinc supplementation can reverse these effects (Wang et al. 2017). Selenium, like vitamin E, is a natural antioxidant that is highly associated with several intracellular antioxidant enzymes, especially GPX. GPX is a glutathione enzyme, an antioxidant enzyme containing selenocysteine, which uses glutathione as a substrate to catalyze hydrogen peroxide and lipid peroxides, mitigating oxidative stress and playing an important role in the antioxidant‐oxidant balance (Schomburg 2011). Due to the long‐term high blood glucose environment, many AGEs are produced, promoting ROS generation and leading to increased oxidative stress. Diabetic mice fed with selenium chow, which upregulates the expression of GPX1 in mouse hearts, significantly improved heart function, reduced myocardial apoptosis, and significantly decreased the expression of DNA methylation protein DNMT2. After in vivo administration of the DNA methylation inhibitor AZA (5‐aza‐2′‐deoxycytidine), the study found that AZA had a similar effect to that of a selenium‐supplemented diet, reducing the DNA methylation rate of the GPX1 gene. This suggests that the selenium‐supplemented diet restored the antioxidant‐oxidant balance by inhibiting DNMT2‐mediated methylation of the GPX1 promoter. (Zhu et al. 2022). Under high‐glucose and high‐fat conditions, mitochondrial dysfunction is one of the reasons for the excessive production of ROS, indicating that the balance between mitochondrial fission and fusion is disrupted (Rovira‐Llopis et al. 2017). Selenium supplementation decreases mitochondrial fission and increases mitochondrial fusion, and the levels of antioxidative enzymes (SOD and catalase) and fat metabolism genes (PPARα and PGC‐1α) are elevated (Mushtaq et al. 2021). The above research shows that a diet supplemented with trace elements may be a dietary therapy for preventing DCM in the future.

Despite promising therapeutic effects of zinc supplementation observed in animal models, a recent double‐blind RCT involving 72 T2DM patients (aged 30–60 years, without baseline zinc deficiency) examined the impact of 220 mg zinc sulfate (containing 50 mg elemental zinc) administered twice weekly for 12 weeks, and did not show significant benefit for weight, blood pressure, and glycemic control (Sayadi et al. 2025). This suggests that such supplementation may not reduce the risk of cardiovascular disease in this population. In addition, higher intake of trace elements does not equate to greater benefits for the human body, as these elements are maintained in a homeostatic balance within the organism. Under physiological conditions, these ions are regulated in strict homeostasis. Once this balance is disrupted, a spectrum of disorders may ensue (Jiang et al. 2025). Accumulating evidence from recent investigations indicates that supra‐physiological zinc supplementation is associated with an increased risk of cardiovascular disorders. Zinc overload induces competitive inhibition with other vital trace elements (e.g., copper and manganese), which in turn impairs the catalytic activity of Cu/Zn SOD. Such inhibitory effects promote the accumulation of peroxide free radicals, enhance oxidative stress responses, and perturb the homeostasis of antioxidant enzyme systems (Das et al. 2025). Ultimately, this effect promotes the occurrence and progression of diseases such as atherosclerosis (Karberg et al. 2022; Kitala et al. 2023). Likewise, the administration of selenium supplements warrants careful consideration. This is because supranutritional selenium supplementation may induce impairment of hepatic metabolic function (Wang et al. 2014; Liu et al. 2025). Although research on zinc and selenium overload in DCM remains limited, oxidative stress is recognized as a key pathological process of this disease. Thus, the suitability of trace element‐supplement dietary interventions as adjunctive therapy for DCM hinges on an evaluation of the patients' baseline nutritional status with respect to trace elements. However, more studies are required to explore the optimal dosage of zinc supplementation.

Furthermore, the requirements and metabolism of trace elements can vary considerably depending on gender (Eroglu et al. 2025), age, genetic background (e.g., polymorphisms in genes related to metal ion metabolism), and the type of diabetes. While the deficiency of certain trace elements has been linked to disease progression, the efficacy of supplementation in preventing or mitigating DCM remains uncertain. Therefore, it is imperative to conduct more rigorously designed randomized controlled trials to provide robust evidence supporting targeted interventions.

The details of the diets supplemented with trace elements are summarized in Table 3.

**TABLE 3:** The role of trace elements on DCM.

Component	Research object	Method	Effect	Mechanism
Zinc^a^ (Gu et al. 2018)	db/db mice	rodent diet (90 mg/4057 kcal) for 3 months	EF↑, FS↑, MT↑, P‐Akt2/Akt2↑, P‐GSK3β/GSK3β↑, P‐GS/GS↑, HK‐II↑, PGC‐1α↑, TRB3↓	Upregulate the expression of MT protein, and improve Akt2‐mediated insulin signaling
Zinc^a^ (Wang et al. 2017)	db/db mice	a normal diet with different amounts of Zn (10/30/90 mg/4057 Kcal) for 6 months	Blood glucose↓, EF↑, ANP↓, p‐p38↓, TNF‐α↓, IL‐1β↓, 3‐NT↓, 4‐NHE↓, Nrf2↑, CAT↑, NQO1↑	Mitigate oxidative stress
Selenium^a^ (Zhu et al. 2022)	SD rats, induced by AGE‐BSA	Feed with contained selenium chow (0.1 mg/kg) for 20 days	LVDP↓, LVSP↑, BNP↓, ROS↓, c‐caspase3↓, DNMT2↓, GPX1↑	Restored antioxidant‐oxidant balance
Selenium^a^ (Mushtaq et al. 2021)	SD rats, alloxan induced	Gavage 0.6 mg/kg selenium (dissolved in 2.5% DMSO) for 11 days	blood glucose↓, HW/BW, HW/TL, fibrosis area↓, GATA4↓, ROS↓, SOD↑, GSH↑, catalase↑, PGC‐1α↑, PPARα↑, Drp1↓, Mfn2↑	Improvement of mitochondrial dysfunction

^a^ Interventional studies.

### 2.4 Natural Dietary Antioxidants

In addition to supplying essential nutrients, fruits and vegetables in our daily diet are rich in natural dietary antioxidants (Table 4). Although these natural antioxidants are not essential in the same manner as vitamins and trace elements, they contribute to maintaining body health and improving the state of diseases. Structurally, these antioxidants are mostly polyphenolic compounds, including resveratrol, anthocyanins, and polyphenols. This section primarily examines polyphenolic dietary antioxidants.

**TABLE 4:** The role of natural dietary antioxidants on DCM.

Component	Research object	Method	Effect	Mechanism
Resveratrol^a^ (Fang et al. 2018)	Male Sprague–Dawley rats, HFD + STZ induced	Gavage resveratrol 50 mg/kg/day/16 weeks	LVEF↑, LEFS↑, E/A ratio↑, ANP↓, BNP↓, β‐MHC↓, SOD↑, MDA↓, SIRT1↑, PGC‐1α↑, PGC‐1α acetylation↓	Activate SIRT1 and increased PGC‐1α deacetylation
Resveratrol^b^ (Fang et al. 2022)	Male Sprague–Dawley rats, HFD + STZ induced	Gavage resveratrol 50 mg/kg/day/16 weeks	ADMA↓, NOS↓, PRMT1↓, DDAH2↑, SIRT1↑, GCN5↓, PGC‐1α↑, PGC‐1α acetylation↓	Inhibit the accumulation of ADMA, suppresses ADMA ‐induced PGC‐1α acetylation
Resveratrol^b^ (Fu and Hao 2025)	80 patients were aged between 65 and 80 years, diagnosed with T2DM for more than 5 years, and had evidence of cardiac dysfunction related to DCM	RES intervention (800 mg per day) for 6 months	LDL‐C↓, FBG↓, TNF‐α↓, IL‐6↓ and LDH↓, HDL‐C↑	Reduce risk indicators for DCM
Resveratrol^a^ (Alanazi et al. 2024)	Male Wistar rats, STZ induced	Gavage resveratrol 40 mg/kg/day/5 weeks	LDH↓, IL‐6↓, TNF‐α↓, NF‐κB↓, MDA↓, CAT↑, GPx↑, caspase3↓, Bax↓, Bcl2↑	Antioxidant and anti‐inflammation effects
Anthocyanin^a^ (Chen et al. 2016)	Male Wistar rats, STZ induced	Gavage anthocyanin 250 mg/kg/day/4 weeks	COX‐2↓, TLR4↓, p‐NFκB↓, IL‐6↓, ANP↓, p‐MEK5↓, LVEDD↑, LVESD↑, fibrosis area↓, MMP‐9↓, TGF‐β1↓, CTGF↓, FGF2↓, p‐ERK1/2↓	Attenuate cardiac hypertrophy and fibrosis
Anthocyanin^a^ (Huang et al. 2017)	Male Wistar rats, STZ induced	Gavage anthocyanin 250 mg/kg/day/4 weeks	TUNEL‐positive cells↓, Fas↓, Caspase‐3/8/9↓, Bad↓, Bak↓, Cytochrome c↓, IGF‐1↑, p‐IGF‐1R↑, p‐Akt↑, Bcl‐2↑, Bcl‐xL↓, p‐Bad↓, EF↑, FS↑	Inhibit cardiomyocyte apoptosis and promote IGF‐1R/PI3K/Akt survival signaling
Anthocyanin^a^ (Yue et al. 2020)	Male C57BL/6 mice, STZ induced	Gavage anthocyanin 250 mg/kg/day/ 12 weeks	LVEF, LVFS, IL‐6, IL‐1β, collagen I, collagen III, IL17, miR‐214‐3p,	Inhibit IL‐17‐related inflammation and fibrosis
Curcumin^a^ (Guo et al. 2018)	Male Sprague–Dawley rats, HFD + STZ induced	Gavage Curcumin 300 mg/kg/day/ 16 weeks	collagen I↓, collagen III↓, TGF‐β↓, TβR2↓, P‐smad2/3↓, samd7↑, p‐AMPKα↓, p‐MAPK↓	Inhibit TGF‐β1/Smad and AMPK/p38 MAPK signaling
Curcumin^a^ (Ren et al. 2020)	Male Sprague–Dawley rats, HFD + STZ induced	Gavage Curcumin 100 mg/kg/day/ 12 weeks	LVEF↑, LVFS↑, fibrosis area↓, SOD↑, MDA↓, TUNEL‐positive cells↓, Cleaved‐caspase‐3↓, Bax↓, Bcl‐2↑, Sirt1↑, PI3K↑, p‐Akt↑, Ac‐Foxo1↓	Regulate Sirt1‐Foxo1 and PI3K‐Akt signaling pathways
Curcumin^a^ (Wei et al. 2022)	Male New Zealand rabbits, STZ induced	Gavage Curcumin 300 mg/kg/day/3 months	Nuclear Nrf2↑, TUNEL‐positive cells↓, GPX4↑, ROS↓, HO‐1↑, COX1↓	Enhance oxidative ability through promoting the nuclear translocation of Nrf2
Curcumin^a^ (Wu et al. 2022)	Male Sprague–Dawley rats, HFD + STZ induced	Gavage Curcumin 100 mg/kg/day/ 12 weeks	EF↓, fibrosis area↓, ROS↓, Nrf2↑, HO‐1↑, Caspase‐3↓, Bax↓, Bcl‐2↑	Alleviate oxidative stress by activating Nrf2/HO‐1 signaling pathways

^a^ Interventional studies.

^b^ Clinical studies.

Resveratrol (RSV) is a naturally occurring non‐flavonoid polyphenolic compound prevalent in various fruits and medicinal plants. The main dietary sources of RSV include grapes, peanuts, blueberries, and mulberries, with red grapes and red wine being the predominant sources (Tian and Liu 2020). In addition to its core anti‐inflammatory and antioxidant effects (Meng et al. 2021), RSV has been reported to improve metabolic disorders (Barber et al. 2022) and exhibit antitumor effects (Talib et al. 2020). In recent years, research has focused on the application of RSV in DCM. For example, after 16 weeks of oral RSV treatment, DCM mice exhibited significant improvements in systemic antioxidant capacity and cardiac dysfunction (Fang et al. 2018). These beneficial effects are closely linked to peroxisome proliferator‐activated receptor gamma coactivator‐1 (PGC‐1α). PGC‐1α is highly expressed in energy‐demanding tissues, such as the heart, skeletal muscle, and brown adipose tissue. It regulates mitochondrial biogenesis, energy metabolism, oxidative stress, and inflammatory responses (Qian et al. 2024). In DCM mice, cardiac PGC‐1α expression is reportedly reduced, and its acetylation level is elevated. Further investigation revealed that asymmetric dimethylarginine (ADMA) levels were significantly increased in the hearts of DCM mice. Exogenous ADMA significantly enhanced PGC‐1α acetylation and induced mitochondrial dysfunction by suppressing SIRT1 (deacetylase), thereby upregulating GCN5 (acetyltransferase) and increasing PRMT1 (ADMA‐synthesizing enzyme). Conversely, all ADMA‐mediated effects were effectively counteracted by RSV (Fang et al. 2022). Additionally, in a clinical study that enrolled 80 T2DM patients with DCM, 800 mg/day of RSV was administered for 6 months. The results showed that RSV treatment significantly reduced cardiovascular risk indicators (LDL‐C, FBG, TNF‐α, IL‐6, and LDH) (Fu and Hao 2025). These findings align with animal experiments, which demonstrated that RSV improved cardiac function. Nevertheless, cardiac function assessments in the patients were not performed.

Although resveratrol has shown promising cardioprotective effects in rodent studies, clinical evidence supporting these benefits is lacking. For example, in a trial of 29 overweight elderly subjects randomized to 300 mg, 1000 mg, or placebo for 90 days, investigators measured changes in cardiovascular risk biomarkers. Surprisingly, the 1000 mg group exhibited an increase rather than a decrease in several risk biomarkers, whereas the 300 mg and placebo groups showed no significant alterations (Mankowski et al. 2020). These findings raise the possibility that high‐dose resveratrol may exert adverse cardiovascular effects in this population. Limitations of the study include the small sample size and the fact that most biomarkers changed only directionally without reaching statistical significance. Furthermore, another limitation stems from the absence of echocardiography, endothelial function testing, or arterial stiffness assessments in this trial. Despite its promising pharmacological activities, RSV is limited by its poor pharmacokinetic properties, which compromise its therapeutic efficacy (Ren et al. 2021). Therefore, improving its drug ability through approaches such as liposomal encapsulation (Alanazi et al. 2024) or structural modification remains challenging.

Anthocyanins are water‐soluble flavonoids, and fruits are their most common dietary source. Fruits containing anthocyanins include apples, pears, berries, stone fruits, and grapes. Moreover, anthocyanins are also found in cabbage and other dark‐colored vegetables. In recent years, the protective effects of anthocyanins have been implicated in DCM. This is primarily achieved by attenuating inflammation and apoptosis and inhibiting fibrosis.

In a rat model of STZ‐induced DCM, anthocyanins suppressed the activation of the NF‐κB signaling pathway and decreased the production of inflammatory mediators, such as COX‐2 and IL‐6, thereby attenuating myocardial inflammation. Furthermore, anthocyanins downregulate the hypertrophic markers ANP and MEK5, inhibit the fibrosis‐related proteins TGF‐β1, CTGF, and MMP‐9, and reduce collagen accumulation in the myocardium (Chen et al. 2016). This ultimately led to a significant amelioration of the cardiac fibrosis. Anthocyanins also exerted anti‐apoptotic effects. For example, it has been demonstrated that anthocyanin intervention decreases the number of TUNEL‐positive cells, suppresses the Fas/FADD/Caspase‐8 extrinsic apoptotic pathway, and reduces the expression of pro‐apoptotic proteins such as Bad and Bak. Moreover, anthocyanins lower the mitochondrial release of cytochrome c and inhibit the activation of Caspase‐9 and Caspase‐3. Simultaneously, it enhances the levels of anti‐apoptotic proteins Bcl‐2 and Bcl‐xL (Li et al. 2018; Huang et al. 2017), thereby promoting cardiomyocyte survival and improving cardiac contractile function.

Recent molecular studies have revealed a novel mechanism by which anthocyanins modulate miRNA expression. By suppressing IL‐17 production in cardiac tissue, anthocyanins decrease the expression of IL‐1β, TNF‐α, and IL‐6. It further diminishes collagen I and III deposition and alleviates myocardial fibrosis. In high‐glucose‐stimulated cardiac fibroblasts, anthocyanins similarly inhibit IL‐17 and attenuate cellular injury and decrease miR‐214‐3p, which regulates IL‐17 (Qi et al. 2020). Transfection with anti‐miR‐214‐3p oligonucleotides markedly blunted the protective effects of anthocyanins. This indicates that the miR‐214‐3p/IL‐17 axis may be a new therapeutic target for DCM (Yue et al. 2020).

Current studies suggest that supplemental anthocyanin intake has a positive effect on cardiovascular diseases, which is mainly attributed to its antioxidant capacity (Yan and Li 2024). However, previous research primarily focused on the role of anthocyanin supplementation in reducing cardiovascular risk or influencing biomarkers, while overlooking the impact of quantitative long‐term anthocyanin consumption on cardiovascular health. In addition, some studies have reported that anthocyanin intake may have no significant effect on cardiovascular health (Lai et al. 2015; Jacques et al. 2015). This reminds us that more clinical evidence is needed to verify the beneficial role of anthocyanins in cardiovascular diseases.

Curcumin, a diketone polyphenol primarily derived from the turmeric plant, is a key component of the spice curry (Kotha and Luthria 2019). Beyond its culinary uses, turmeric has been documented for its medicinal value in traditional texts from India and China. With modern purification techniques, curcumin has been isolated, further expanding its application. Its potent anti‐inflammatory and antioxidant properties have shown potential for improving DCM. For instance, in both in vivo and in vitro, curcumin was shown to promote nuclear translocation of Nrf2, thereby upregulating downstream antioxidant factors such as HO‐1 and GPX4. This resulted in decreased ROS levels, thereby suppressing high‐glucose‐induced ferroptosis in cardiomyocytes. Moreover, curcumin reversed myocardial injury evoked by the ferroptosis inducer erastin, indicating that its cardioprotective effect is mediated through the activation of the Nrf2 signaling pathway (Wei et al. 2022). Wu et al. (2022) further demonstrated that the antioxidant effect of curcumin is dependent on the Nrf2/HO‐1 pathway. Similar to other natural antioxidants, curcumin has anti‐apoptotic and anti‐myocardial fibrosis effects. For example, curcumin protects the myocardium by activating Sirt1 expression, suppressing Foxo1 acetylation, and enhancing Akt phosphorylation, which ultimately inhibits apoptotic pathways (Ren et al. 2020). Moreover, it markedly reduces type I and type III collagen deposition and alleviates cardiac fibrosis in diabetic rats by blocking the classical profibrotic TGF‐β1/Smad signaling cascade (Meng et al. 2016; Guo et al. 2018).

However, the application of curcumin is limited by its structural instability and low bioavailability (Nelson et al. 2017; Abd El‐Hack et al. 2021). At present, curcumin treatment has also failed to demonstrate significant cardiovascular benefits, possibly due to its low bioavailability, especially when administered at low doses (Hewlings and Kalman 2017). Fortunately, recent approaches have emerged to enhance curcumin's therapeutic performance, including direct structural modification (Xiang et al. 2020), nano‐complexing (Rahiman et al. 2022), and complex coacervation. Therefore, for the clinical application of curcumin, it is still necessary to address its low bioavailability and determine the dietary dosage required to achieve a therapeutically effective level.

Although these natural antioxidants have shown beneficial effects on diabetic cardiac function in animal studies, the scarcity of clinical data limits their therapeutic application, particularly regarding optimal dosage, treatment duration, and potential drug interactions. Furthermore, current research on natural antioxidants is still predominantly based on animal experiments, primarily exploring their potential mechanisms of action. For clinical application, large‐scale RCTs are still needed to demonstrate the true benefits of natural antioxidants for the human body. Given that these compounds are naturally present in daily foods and dietary intake from fruits and vegetables remains far below the doses used in trials (e.g., Resveratrol 30–150 mg (Xu et al. 2024), Anthocyanins: 80 mg/day (Guo, Zhang, Liu, et al. 2020), Curcumin: 0–3 mg/kg/day (Kumar, Harsha, et al. 2021)). As such, increasing consumption of a diverse range of antioxidant‐rich foods is still recommended.

### 2.5 The Role of Dietary Patterns on DCM

#### 2.5.1 Ketogenic Diets

A KD, which is very low in carbohydrates, high in fat, and moderate in protein, reduces carbohydrate intake and increases ketone body levels in the blood. It has been widely used as an adjunctive therapy for children with drug‐resistant epilepsy (Ulamek‐Koziol et al. 2019) and has also demonstrated positive effects in managing diabetes and promoting weight loss (Kumar, Behl, et al. 2021). In recent years, the role of the KD in diabetic complications has been studied. Ketone bodies, which are fat metabolites, can be used as an alternative energy source when the supply of glucose is insufficient. As blood ketone levels increase, insulin secretion decreases. The decrease in insulin secretion and increase in glucagon levels stimulate fat decomposition in the liver and adipose tissue to produce more ketones (Kleissl‐Muir et al. 2022).

β‐Hydroxybutyric acid (BHB) is a ketone body produced by fat metabolism. Following a KD, BHB levels in the body are notably elevated owing to the increased metabolism of fats for energy, which enhances cellular antioxidant capacity (Oka et al. 2021). The study found that under a KD, the heart enhances the utilization rate of ketone bodies and reduces its oxidative dependence on fatty acids and glucose, which is likely related to the upregulation of p‐Akt/Akt (Trang et al. 2023). The PI3K‐Akt pathway regulates various biological processes, including metabolism, proliferation, migration, and survival (Ghigo et al. 2017). Under physiological conditions, insulin binds to its receptor (INSR), resulting in the phosphorylation of insulin receptor substrate (IRS) proteins. This, in turn, activates class I PI3K (particularly the p110α subtype) (Savova et al. 2023), thereby promoting glucose uptake, glycogen synthesis, and gluconeogenesis suppression. However, when the body develops insulin resistance, various factors (such as AGEs, elevated free fatty acids, and inflammatory factors) inhibit the tyrosine phosphorylation of IRS. Simultaneously, it enhances serine phosphorylation, impairing its ability to effectively activate PI3K. The loss of these protective functions is a direct cause of the development of various diabetic complications (Taheri et al. 2024; Huang et al. 2018). In one study, the content of PI3K and the ratio of p‐Akt/Akt (PI3K receptor) in the hearts of diabetic mice decreased, and these changes were partially reversed by treatment with a KD. In vitro, p‐Akt/Akt was increased in diabetic cardiomyocytes after BHB treatment, whereas caspase‐3, a key enzyme in the apoptotic process, was decreased. This cardioprotective effect of BHB can be blocked by PI3K inhibitors, indicating that a KD protects cardiomyocytes from DCM by activating the PI3K‐Akt pathway (Guo, Zhang, Shang, et al. 2020).

In addition to metabolic balance, calcium homeostasis plays an important role in maintaining normal heart function, as it is involved in the regulation of heart rate and muscle contractions. The precise balance of calcium ions within cardiac cells ensures proper coordination of the cardiac cycle, including both systole and diastole. This balance is achieved through the concerted actions of various proteins and mechanisms (Lee et al. 2023). After KD treatment, diabetic mice exhibited diminished phosphorylation of proteins that are involved in the regulation of calcium ion release in the heart, such as pRyR2/RyR, pCaMKIIδ/CaMKII‐δ, and pPLB‐S16/PLB. Furthermore, the expression of proteins associated with calcium ion influx was increased, including NCX, SERCA2a, and Cav1.2. This effect increased the calcium concentration in myocardial cells, activates myocardial contractions, and improves excitation‐contraction coupling efficiency, which restores the cardiac contractile function impaired by diabetes.

However, in recent years, some researchers indicated that the KD not only has no protective effect on cardiomyocytes of DCM, but also has the opposite effect. In a 62‐week animal study, mice showed reductions in blood glucose, triglyceride, and insulin levels, which seemed to improve diabetes; however, the study found that the mice had myocardial hypertrophy and impaired diastolic function. The authors used 13‐C‐labeled acetoacetic acid, butyrate, and pyruvate to explore heart metabolism in mice with DCM. The myocardial total ketone utilization rate was lower in the KD group than in the chow diet group. Under low‐carbohydrate conditions, the heart upregulates myocardial glycolysis but does not enhance fatty acid oxidation as expected. Although it is still impossible to distinguish whether it is pathological or physiological myocardial hypertrophy, an increase in myocardial lipotoxicity markers was observed in the hearts of KD‐fed diabetic rats, which has a trend toward pathological myocardial hypertrophy (Abdurrachim et al. 2019). In addition, a KD may have other negative effects. Regulatory T cells (Tregs) play an important role in attenuating myocardial fibrosis (Cao et al. 2013; Kanellakis et al. 2011). In a study, the proportions of circulatory Treg cells (CD4^+^, CD25^+^, Foxp3^+^) in whole blood cells were reduced in mice fed with KD, as well as the production of the ST2L ligand, IL‐33, which acts through its receptor ST2, a membrane receptor of the interleukin family. Moreover, MAMs, a key structure that controls mitochondrial respiratory function, decreased in the presence of KD, promoting cardiac fibroblast activation and interstitial fibrosis. However, reinforcement of ST2L in Tregs prevented the loss of MAMs in KD‐treated Treg cell (Tao et al. 2021). Therefore, a KD may promote myocardial fibrosis by blunting IL‐33/ST2L signaling in Treg cells. Additional studies have suggested that a KD may induce myocardial fibrosis (Xu et al. 2021).

**FIGURE 2:**
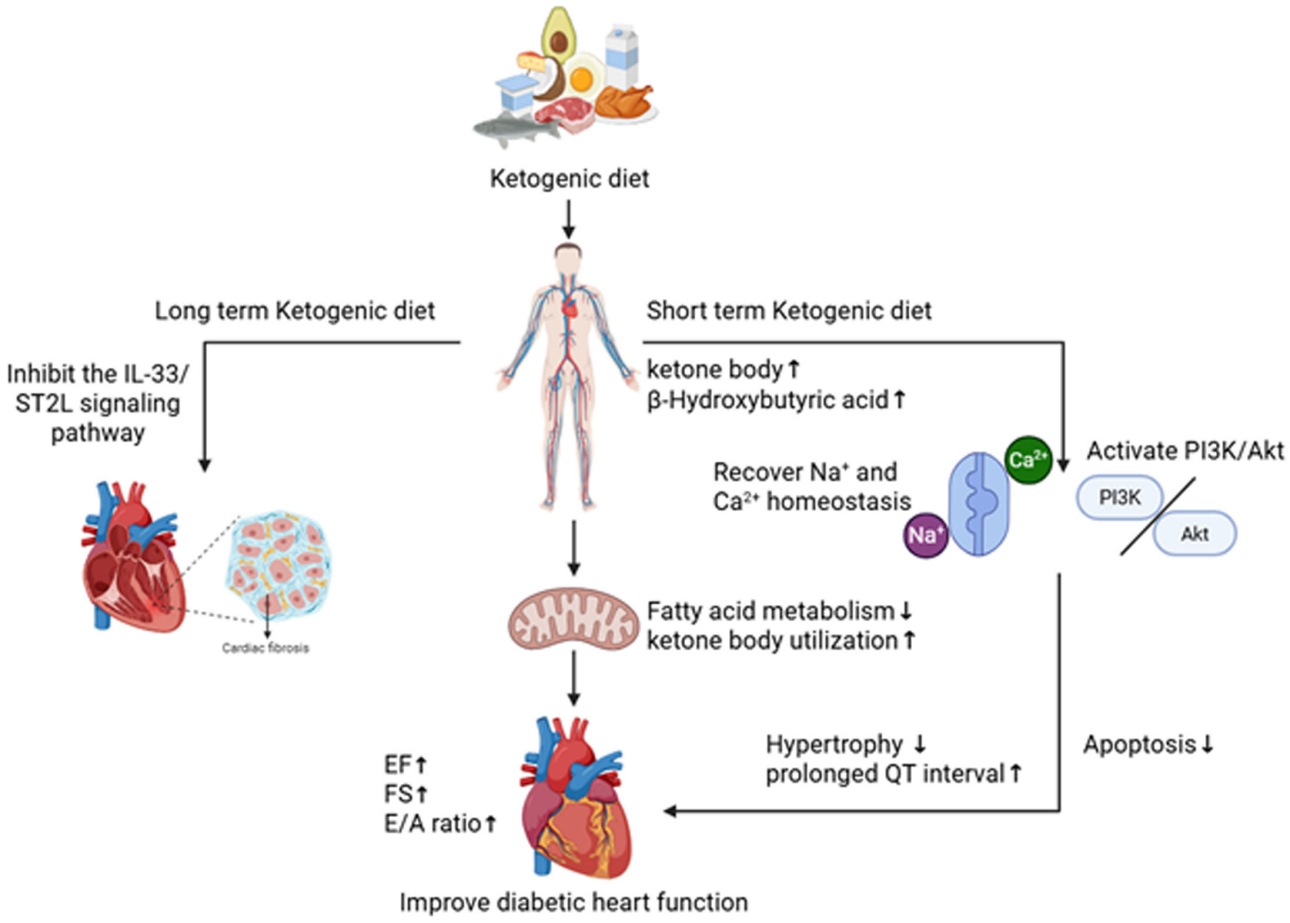
The effect of KD on DCM. A short‐term ketogenic diet may improve diabetic cardiomyopathy by enhancing mitochondrial metabolism, restoring sodium‐calcium ion homeostasis, and activating the PI3K/Akt pathway, whereas a long‐term ketogenic diet may induce myocardial fibrosis by suppressing the IL‐33/ST2L signaling pathway.

Although most studies suggest that a KD is beneficial for good health, it carries certain risks for people with diabetes and can even aggravate DCM. In addition, a KD increases the risk of diabetic ketoacidosis due to elevated blood ketone levels. Furthermore, because KD is only used as an adjuvant therapy in clinical practice, and patients still need to continue medication to a large extent, interactions with contemporary diabetes therapies, such as SGLT2 inhibitors, further increase the risk of ketoacidosis (Koutentakis et al. 2023; Dwyer et al. 2023). In current research on the KD, a few studies demonstrating negative effects are based on long‐term animal experiments lasting up to 62 weeks (Abdurrachim et al. 2019). In contrast, several studies showing protective effects are likely short‐ or medium‐term (6–12 weeks) experiments (Figure 2). This suggests that the benefits of KD may be short‐term, whereas long‐term risks (such as fibrosis and hypertrophy) may emerge over time (more than 12 weeks). Under long‐term (36–44 weeks) KD feeding in mice, initial reductions in blood glucose were observed in the short term. Nevertheless, prolonged exposure resulted in fasting hyperglycemia, pronounced glucose intolerance, and, in males, hepatic steatosis accompanied by liver dysfunction (Gallop et al. 2025). Duration is likely a critical variable in assessing the cardiac safety of the KD. There is an urgent need for well‐designed long‐term human clinical trials utilizing cardiac imaging (e.g., cardiac MRI) and biomarkers to comprehensively evaluate the impact of KD on cardiac structure and function in patients with diabetes mellitus. Moreover, further research is required to elucidate why cardiac metabolic responses (to fatty acid and ketone utilization) differ across studies. Based on the current evidence, the KD should not be regarded as a suitable long‐term strategy for health promotion. Additional studies are essential to refine its safety profile, especially in populations with pre‐existing metabolic conditions.

The details of the KD are summarized in Table 5.

**TABLE 5:** The role of ketogenic diet on DCM.

Dietary strategies	Research object	Dietary composition	Effect	Mechanism
Ketogenic diet^a^ (Trang et al. 2023)	SD rats, STZ induced	43% protein, 38.4% fat 7.8% fiber, and 2.8% carbohydrates for 8 weeks	Blood glucose↓, TG↓, TC↓, LDL↓, HDL↑, EF↑, FS↑, fibrosis area↓, BDH1↑, CPT‐1β↑, pAMPKα2/AMPKα2↑, CD36↑, PERK↑, p‐eIF2α↑	Augment ketone body metabolism, reduce FA oxidation and endoplasmic reticulum stress
Ketogenic diet^a^ (Guo, Zhang, Shang, et al. 2020)	db/db mice	18.2% protein and 66.5% fat chow for 8 weeks	Blood glucose↓, EF↑, cardiac fibrosis↓, TG↓, Lopa1/Sopa1↑, ROS↓, MDA↓, MnSoD↑, caspase‐3↓, Bcl‐2/Bax↑, PI3k↑, P‐Akt/Akt↑	Attenuate myocardial apoptosis through PI3K/Akt pathway
Ketogenic diet^a^ (Lee et al. 2023)	Wistar rats, STZ induced	65.3% fat, 2% carbohydrates, and 32.6% protein for 6 weeks	EF↑, FS↑, intracellular calcium transients↑, decay time of intracellular calcium↓, QTc interval ↓, action potential duration↓, I_Na‐Late_↓, Na^+^/H^+^ exchanger↑, ROS↓, Na^+^↓, pCaMKII‐δ/CaMKII‐δ↓, SERCA2a↑	Attenuate the effects of DCM‐dysregulated Na^+^ and Ca^2+^ homeostasis
Ketogenic diet^a^ (Abdurrachim et al. 2019)	Male lean diabetic Goto‐Kakizaki (GK/MolTac) rats	93.5 kcal% fat, 4.7 kcal% protein, 1.8 kcal% carbohydrate for 62 weeks ad libitum.	β‐OHB↑, free fatty acids↑, ANP↑, HW/BW↑, Cardiomyocyte size↑, myocardial ketone body oxidation↓, SCOT↓, BDH1↓, ACADL↑, HADH↑	Maladaptive cardiac metabolic modulation and lipotoxicity
Ketogenic diet^a^ (Tao et al. 2021)	db/db mice	KD (10% protein, < 1% carbohydrate, and 89% fat) for 12 weeks	Fasting insulin↑, HOMA‐IR↑, −dp/dtmax↓, E/A ratio↓, collagen volume fraction↑, IL4↓, IL10↓, IL33↓, ST2↓	Activate cardiac fibroblasts and interstitial fibrosis by inhibiting the IL‐33/ST2L signaling pathway

^a^ Interventional studies.

#### 2.5.2 Caloric Restriction

Calorie restriction (CR) is a restricted diet that reduces energy intake by about 25%–30% without affecting the intake of essential nutrients (Napoleao et al. 2021). Many studies have shown that caloric restriction can provide considerable benefits to humans. Studies on caloric restriction in some non‐human primates and humans have preliminarily shown that it can prolong the life span and prevent age‐related diseases (Roth and Polotsky 2012). At present, researchers are exploring other benefits of caloric restriction, such as limiting tumor growth (Pomatto‐Watson et al. 2021) and antidepressant effect (Manchishi et al. 2018). Endogenous cannabinoids can promote energy intake and storage. When the endogenous cannabinoid system becomes overactive, it may impair both glucose and fat metabolism and even influence diabetes‐induced oxidative stress, inflammation, and fibrosis (Gruden et al. 2016). The two main forms of endogenous cannabinoids are anandamide (AEA) and 2‐arachidonoyl glycerol (2‐AG) (Horvath et al. 2012). In a prospective intervention study, patients with T2DM and coronary artery disease were given a caloric restriction diet (450–1000 kcal/day) for 16 weeks. After 16 weeks of CR intervention, there was a decrease in AEA levels and a reduction in subcutaneous white adipose tissue, epicardial adipose tissue, and paracardial adipose tissue; these effects were accompanied by an increase in the left ventricular ejection fraction of the patients. The results indicated that the reduction of AEA may help to improve the metabolic phenotype caused by weight loss and improve the heart function of patients with diabetes (van Eyk et al. 2018).

Peroxisome proliferator‐activated receptor α is involved in fatty acid metabolism in tissues with high oxidation rates, such as the muscle, heart, and liver (Pawlak et al. 2015). In a calorie‐restricted diet study, CR increased the expression of PPARα and decreased free fatty acids in the blood and inflammatory markers in the myocardium (TLR2, TLR4, TNFα, and FetA) (Cohen et al. 2017). FetA is an endogenous ligand of TLR4, and its binding induces the release of inflammatory factors, leading to FFA‐induced insulin resistance (Pal et al. 2012). CR may prevent heart damage through two mechanisms: First, CR upregulates PPARα levels, which reduces free fatty acids by enhancing fatty acid metabolism. In contrast, CR inhibits the expression of TLR2 and TLR4, which decreases FFA uptake and FetA levels, attenuates cardiac inflammation, and subsequently improves insulin resistant (Cohen et al. 2017).

SIRT1 is an important target for reducing the progression of diabetic cardiomyopathy. SIRT1 is a conserved NAD^+^‐dependent protein closely related to cellular metabolism. It can avoid metabolic pressure by deacetylating target proteins in tissues and organs, such as the liver, heart, and adipose tissue (Chang and Guarente 2014). Once SIRT1 is induced, it interacts with and deacetylates PGC‐1α in an NAD^+^‐dependent manner (El‐Khamisy et al. 2005). PGC‐1α serves as a PPARγ coactivating factor, regulates mitochondrial DNA replication, respiratory chain gene expression, fatty acid oxidation, and antioxidant response, and its expression and activity are regulated by nutritional status (obesity/CR) (Kobayashi et al. 2021). The expression of PGC‐1α is inhibited in metabolic diseases, and activation of PGC‐1α promotes mitochondrial function and energy expenditure to combat metabolic diseases, such as obesity and nonalcoholic fatty liver disease (Wu et al. 2020; Liao et al. 2024; Leng et al. 2022). Maayan Waldman's research showed that the CR has the same effect in regulating fatty acid metabolism and improving mitochondrial function by activating SIRT1‐PGC1α (Planavila et al. 2011). Further research found that the expression of HO‐1 increased in the calorie restriction diet, and HO‐1 participated in the SIRT1‐PGC‐1α axis. HO‐1 is an important antioxidant enzyme that is regulated by a variety of cellular pathways. To verify the role of HO‐1 in the SIRT1‐PGC‐1α axis, the study used an HO‐1 inhibitor, which decreased the expression of SIRT1 and PGC‐1α. After the addition of the HO‐1 activator, the expression of SIRT1 and PGC‐1α increased and improved heart function, which indicates that HO‐1 participates in the SIRT1‐PGC‐1α axis and plays an important role in protecting the diabetic heart (Waldman et al. 2019).

**FIGURE 3:**
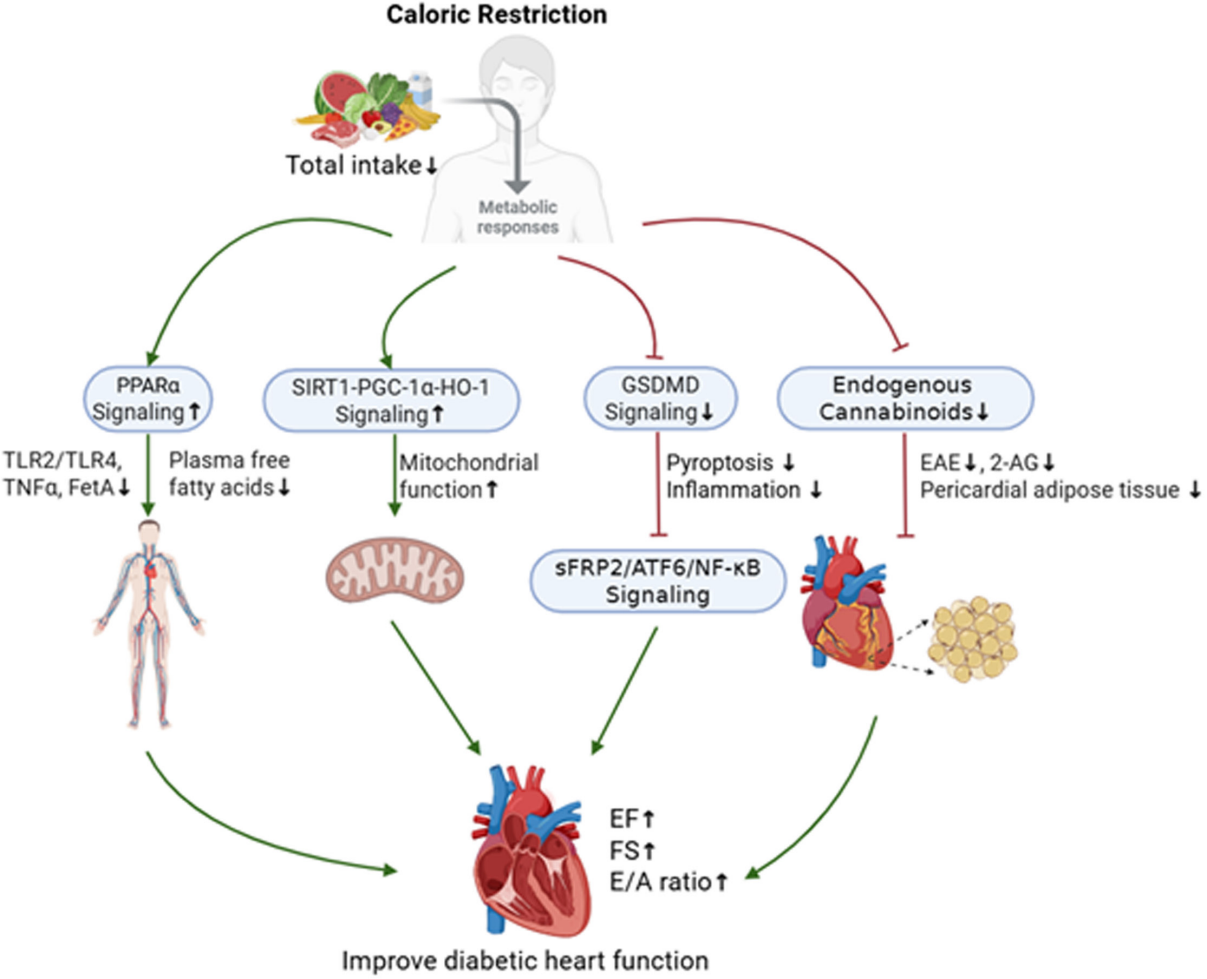
The effect of CR on DCM. Caloric restriction exerts cardioprotective effects in diabetic cardiomyopathy primarily by reducing energy intake, which lowers endogenous cannabinoid levels (AEA) to alleviate lipotoxicity, activates the PPARα pathway to enhance fatty acid oxidation and reduce inflammation, upregulates the SIRT1‐PGC‐1α‐HO‐1 axis to improve mitochondrial biogenesis and antioxidant defense, and inhibits the GSDMD‐SFRP2‐ATF6‐NF‐κB pathway to attenuate cardiac inflammation and pyroptosis.

In addition, a recent study demonstrated that CR attenuated diabetic cardiac inflammation induced by GSFMD, upregulating the SFRP2/Atf6/NF‐ΚB pathway. Deletion of GSDMD mimicked the cardioprotective effects of CR. At the same time, cardiomyocyte‐specific overexpression of GSDMD counteracted the beneficial effects of CR, whereas exogenous supplementation of recombinant SFRP2 reversed the cardioprotective effect of GSDMD deletion (Lin, Zhai, et al. 2025). Therefore, CR may exert cardioprotective effects through multiple signaling pathways. These pathways include improving metabolism and mitochondrial function through the SIRT1‐PGC‐1α‐HO‐1 axis and reducing inflammation and necrosis by inhibiting the GSDMD‐SFRP2‐ATF6‐NF‐κB pathway. However, future research must focus on the mechanisms that explain how caloric restriction improves DCM. Fortunately, current research approaches are increasingly focused on identifying the key metabolites responsible for the benefits of caloric restriction (Davinelli et al. 2020; Fuerlinger et al. 2025). Utilizing these critical metabolites or their mimetics to ameliorate diseases offers a novel therapeutic strategy (Hofer et al. 2021; Madeo et al. 2019), thereby providing an alternative for patients who are clinically unable to adhere to caloric restricted diets. The multi‐pathway cardioprotective mechanism of caloric restriction in diabetic cardiomyopathy is illustrated in Figure 3, which systematically depicts the key signaling pathways and molecular targets of CR in alleviating myocardial injury, oxidative stress and inflammation.

Clinical evidence, though limited, supports these findings. Recent meta‐analyses further substantiate CR's benefits. For example, Jayedi et al.'s (2023) study (28 RCTs, 6281 T2DM patients) showed CR increases remission (HbA1c < 6.5% without meds: 38 more/100 at 6 months), with dose–response effects: each 500 kcal/day reduction lowers weight (−6.33 kg) and HbA1c (−0.82%) by 12 months, especially with intensive lifestyle programs. In addition, Semnani‐Azad et al.'s (2025) meta‐analysis (99 RCTs, 6582 adults) compared intermittent fasting (IF) variants with continuous CR, finding all reduce weight vs. ad libitum, with alternate‐day fasting modestly superior short‐term (−1.29 kg vs. CR) for weight and lipids. Schroor et al.'s (2024) meta‐analysis (28 RCTs, 2043 healthy adults) revealed intermittent energy restriction (IER) comparable to continuous CR for weight/fat loss, but IER further reduces fat‐free mass (−0.20 kg) and waist circumference (−0.91 cm), with subgroup benefits (e.g., time‐restricted eating lowers fat more). These syntheses highlight CR's short‐term efficacy in T2DM, potentially mitigating DCM via reduced hyperglycemia and lipotoxicity, though long‐term human trials targeting DCM endpoints are needed to show direct heart benefits.

However, despite these promising mechanisms and benefits observed in preclinical and small clinical studies, caloric restriction faces several practical challenges in patients with diabetes. Long‐term adherence is a significant challenge in implementing CR in daily life, often influenced by work, life, emotions, and calorie‐tracking habits. Several studies have shown that both older adults and younger individuals exhibit high adherence and feasibility to CR (> 80%), with no significant age‐related infeasibility (Fanning et al. 2025; Hegedus et al. 2024; Wang, Wang, et al. 2025; Park et al. 2025; Lin, Ezzati, et al. 2025). However, younger adults demonstrate slightly better adherence (higher satisfaction and retention rates), while older adults require more remote support and simplified monitoring. These differences may stem from lifestyle factors (younger adults have more flexible schedules, while older adults have more routine ones) and perceived burden (older adults find logging records more burdensome). Nevertheless, these studies also reveal certain limitations: small sample sizes and relatively short monitoring periods (longest 9 months, shortest 8 weeks). Additionally, adherence tends to decline over time (Fanning et al. 2025). Unresolved questions remain, such as how long CR needs to be monitored to observe benefits and whether discontinuing calorie restriction leads to weight rebound. These questions suggest that current evidence preliminarily supports the feasibility of CR, but larger‐scale, long‐term, multi‐center studies are needed to address the aforementioned problems.

Additionally, prolonging CR may increase the risk of sarcopenia (age‐related muscle loss) and osteoporosis in elderly patients (Batsis et al. 2017; Jiang and Villareal 2019). However, existing studies have shown that, based on caloric restriction, adding exercise (especially aerobic plus resistance exercise) could improve muscle protein synthesis and muscle quantity (Colleluori et al. 2019; Jiang and Villareal 2025). If caloric restriction (CR) is recommended as an adjuvant therapy for elderly patients with DCM in clinical practice, it will also be necessary to require them to perform aerobic plus resistance exercise. Like KDs, calorie‐restricted diets necessitate evaluation of potential drug‐diet interactions. Research on this combination remains limited, with the majority of existing studies highlighting the benefits of SGLT2 inhibitors alongside calorie restriction. Additional clinical evidence is required to establish the long‐term safety of this approach. Clinical trials also highlight variability in response, with benefits attenuating over time without ongoing support. Thus, CR should be individualized, supervised, and ideally integrated with behavioral interventions to maximize sustainability and minimize risks.

The details of caloric restriction are summarized in Table 6.

**TABLE 6:** The role of caloric restriction on DCM.

Dietary strategies	Research object	Dietary composition	Effect	Mechanism
Caloric restriction^a^ (Cohen et al. 2017)	db/db mice, angiotensin induced	The mice were fed 90% of their average caloric intake for 2 weeks (10% restriction), followed by 65% of that for additional 2 weeks (35% restriction)	Glucose↓, TC↓, fibrosis area↓, TLR2↓, TLR4↓, TNFα↓, PPARα↑, FFA↓, AKT↓	Upregulate the expression of PPAR‐α, which improves myocardial fatty acid metabolism
Caloric restriction^b^ (van Eyk et al. 2018)	Obese (BMI > 25 kg/m^2^) T2D patients with documented coronary artery disease (27 patients)	400–600 kcal/day (high protein content of 67% and a low fat content of 5%), supplemented with a limited choice of vegetables for 3 weeks, then gradually increasing caloric intake, it reached 1000 kcal/day by the 16th week	Glucose↓, TG↓, TC↓, HDL↑, AEA↓, subcutaneous white adipose tissue↓, epicardial white adipose tissue↓, paracardial white adipose tissue↓, EF↑, E/Ea↓	Reduce endogenous cannabinoid AEA, which reduces lipid accumulation in the heart
Caloric restriction^a^ (Waldman et al. 2018)	db/db mice, angiotensin induced	The mice were fed 90% of their average caloric intake for 2 weeks (10% restriction), followed by 65% of that for additional 2 weeks (35% restriction)	Glucose↓, FS↑, ANP↓, BNP↓, TGF‐β↓, TNF‐α↓, MMP2↓, P‐ERK1/2↓, adiponectin↑, P‐AMPK↑, PPARγ↓, SIRT1↑, PGC‐1α↑	Activate PGC‐1α and improve mitochondrial function
Caloric restriction^a^ (Waldman et al. 2019)	db/db mice, angiotensin induced	The mice were fed 90% of their average caloric intake for 2 weeks (10% restriction), followed by 65% of that for additional 2 weeks (35% restriction)	Glucose↓, FS↑, MDA↓, SIRT1↑, PGC‐1α↑, HO‐1↑, ROS↓	Activate SIRT1‐PGC‐1α‐HO‐1 Axis and reduce oxidative stress
Caloric restriction^a^ (Lin, Zhai, et al. 2025)	C57 mice, HFD + STZ induced	Fed with chow at a calorie intake equal to 30% of calorie intake in week 12 for a week, followed by chow ad libitum for the next a week. Such dietary scheme was repeated afterwards for 4 cycles	CK↓, EF↑, FS↑, E/A ratio↑, TUNEL‐positive cells↓, NLRP3↓, GSDMD↓, IL‐1β↓, ASC↓, ATF6↓, sFRP2↓, p‐NF‐κB/NF‐κB↓	Inhibit GSDMD‐dependent sFRP2/ATF6/NF‐κB pathway

^a^ Interventional studies.

^b^ Clinical studies.

### 2.6 Significance of Diet Adjustment in the Treatment of DCM

The benefits brought about by the adjustment of dietary structure can not only improve the pathological state of the body but also reduce the economic burden on patients. As a chronic disease, diabetes can be treated with drugs for a long time or even a lifetime. Not only does diabetes itself require drug treatment, but many complications caused by diabetes are a thorny problem, which also consumes a large amount of money for patients. However, existing research shows that diet treatment can not only prevent cardiomyopathy in patients with diabetes but also reduce the intake of insulin and oral hypoglycemic drugs in patients with T2DM (Dashti et al. 2021; Obermayer et al. 2023, 2022), reducing the treatment cost of patients and social medical expenses (Herman et al. 2021). The reduction in drug intake can also reduce the possible adverse reactions to drugs, such as hypoglycemia, lactic acid poisoning, and diarrhea.

### 2.7 Limitations and Prospects

The translation of experimental animal results to clinical applications faces several challenges. There is a lack of unified standard dietary protocols. Significant variations exist across studies in dosages, nutrient composition, intervention duration, models of diabetes, and cardiac phenotyping, which severely hamper cross‐study comparisons and meta‐analyses. To improve reproducibility and facilitate evidence synthesis, we propose the minimal reporting standards for diet‐DCM studies. Future research should routinely report the following, at a minimum:

Dietary composition: precise macronutrient ratios (carbohydrate, fat, protein as % of energy), source and dose of key bioactive compounds or supplements, and total caloric intake (if applicable).

Intervention duration: clear distinction between short‐term (≤ 12 weeks), medium‐term (12–24 weeks), and long‐term (> 24 weeks) exposure, given emerging evidence of time‐dependent effects (e.g., KD).

Diabetes model and type: specification of type 1 or type 2 diabetes induction method (e.g., STZ dose, high‐fat diet duration), baseline glycemic control (fasting glucose, HbA1c), and insulin resistance status.

Cardiac phenotype: comprehensive baseline and endpoint assessment using standardized methods (e.g., echocardiography for ejection fraction, diastolic function E/A ratio; cardiac MRI or histology for fibrosis; biomarkers such as NT‐proBNP).

Animal/human cohort details: strain/age/sex (preclinical) or patient demographics, comorbidities, and concomitant medications (clinical).

Adopting such standards would enable better stratification of benefits versus neutral/harmful effects. Furthermore, it would support systematic reviews and accelerate translation into clinical guidelines. Second, implementing dietary interventions alone in clinical applications remains challenging because they are typically combined with medications. Therefore, future trials should systematically evaluate potential interactions between dietary strategies and contemporary pharmacotherapies (e.g., SGLT2 inhibitors, metformin). It is also worth noting that not only is the onset and progression of diseases influenced by genetics, but genetic variations related to obesity, metabolic status, and dietary preferences are also involved (Heianza and Qi 2017; Klotz and Carlberg 2023). Exploring nutrigenomics in DCM may emerge as a future research direction for dietary interventions (Lam et al. 2025; Pena‐Romero et al. 2018).

Dietary adjustment also has problems with individualization and compliance. Not all patients accept dietary treatment. Adhering to a recommended diet is a common problem for patients with diabetes. This increases the risk of various complications. The main reason for this is related to their low cultural, religious, and socioeconomic status (Kumar et al. 2022). At present, the main solutions are dietary education and social support (Albanese et al. 2019; Pratiwi et al. 2024). Developing a simple and feasible diet plan for patients integrated with pharmacotherapy could improve patient compliance.

## 3 Conclusion

The progression of hyperglycemia to DCM is a complex process involving a series of mechanisms, and there is currently no effective treatment for this condition. Therefore, people have focused on diet management and hope to improve DCM through dietary adjustments. It can reduce myocardial lipid accumulation and improve oxidative stress injury and fibrosis in the diabetic myocardium. In summary, dietary adjustment is a non‐drug treatment that can improve the pathological status of DCM and the quality of life of patients.

## Author Contributions

**Chi Shu:** writing – review and editing, conceptualization. **Kai‐xuan Lin:** methodology, data curation. **Wen‐hui Deng:** methodology, software, data curation, writing – original draft. **Yuan Li:** data curation. **Han‐Bin Lin:** conceptualization, writing – review and editing. **Xin‐Yue Tong:** data curation. **Hao‐Dong Cui:** data curation. **Abdallah Iddy Chaurembo:** data curation. **Francis Chanda:** data curation. **Li‐Dan Fu:** data curation.

## Funding

This study was supported by research grants from the National Natural Science Foundation of China (82100391 to H.‐B.L.), the Guangdong Provincial Pearl River Talent Program (211283781015 to H.‐B.L.), the State Key Laboratory of Drug Research of Shanghai Institute of Materia Medica Chinese Academy of Sciences (Grant Nos. SKLDR‐2024‐KF‐08 to H.‐B.L.), the High‐level New R&D Institute (2019B090904008 to H.‐B.L.), and the High‐level Innovative Research Institute (2021B0909050003 to H.‐B.L).

## Ethics Statement

The authors have nothing to report.

## Consent

The authors have nothing to report.

## Conflicts of Interest

The authors declare no conflicts of interest.

## Data Availability

The authors have nothing to report. All data discussed are from previously published sources.
